# Common Principles in Functional Organization of VIP/Calretinin Cell-Driven Disinhibitory Circuits Across Cortical Areas

**DOI:** 10.3389/fncir.2020.00032

**Published:** 2020-06-09

**Authors:** Alexandre Guet-McCreight, Frances K. Skinner, Lisa Topolnik

**Affiliations:** ^1^Krembil Brain Institute - Division of Clinical and Computational Neuroscience, Krembil Research Institute, University Health Network, Toronto, ON, Canada; ^2^Department of Physiology, University of Toronto, Toronto, ON, Canada; ^3^Department of Medicine (Neurology), University of Toronto, Toronto, ON, Canada; ^4^Department of Biochemistry, Microbiology and Bio-informatics, Laval University, Québec, QC, Canada; ^5^Neuroscience Axis, CHU de Québec Research Center (CHUL), Québec, QC, Canada

**Keywords:** hippocampus, cerebral cortex, vasoactive intestinal polypeptide, interneuron, microcircuit, disinhibition, behavior

## Abstract

In the brain, there is a vast diversity of different structures, circuitries, cell types, and cellular genetic expression profiles. While this large diversity can often occlude a clear understanding of how the brain works, careful analyses of analogous studies performed across different brain areas can hint at commonalities in neuronal organization. This in turn can yield a fundamental understanding of necessary circuitry components that are crucial for how information is processed across the brain. In this review, we outline recent *in vivo* and *in vitro* studies that have been performed in different cortical areas to characterize the vasoactive intestinal polypeptide (VIP)- and/or calretinin (CR)-expressing cells that specialize in inhibiting GABAergic interneurons. In doing so, we make the case that, across cortical structures, interneuron-specific cells commonly specialize in the synaptic disinhibition of excitatory neurons, which can ungate the integration and plasticity of external inputs onto excitatory neurons. In line with this, activation of interneuron- specific cells enhances animal performance across a variety of behavioral tasks that involve learning, memory formation, and sensory discrimination, and may represent a key target for therapeutic interventions under different pathological conditions. As such, interneuron-specific cells across different cortical structures are an essential network component for information processing and normal brain function.

## Introduction

Interneuron-specific (I-S) cells are inhibitory interneurons that are specialized to primarily target other interneurons. Most often they are identified via expression of vasoactive intestinal polypeptide [VIP; a neuropeptide that was initially localized in gastrointestinal nerves and ventromedial hypothalamus (Larsson et al., 1976)], and calretinin [CR; a calcium-binding protein (Rogers, 1987)]. Some CR+ cells and VIP+ cells in hippocampus and cortex also express serotonin (5HT) receptor mRNA, including 5HT2, 5HT3A receptor (Lee et al., 2010; Tremblay et al., 2016; Prönneke et al., 2019) and 5HT6 receptor (Helboe et al., 2015). As well, VIP has been shown to modulate the effects of 5HT (Rostene et al., 1983). There is also some indication that some CR+ cells in hippocampus express corticotropin-releasing hormone (Gunn et al., 2019). In RNA sequencing datasets obtained from human middle temporal gyrus, VIP+ cells possess the richest diversity in transcriptomically-defined types when compared to non-VIP+ cells (Hodge et al., 2019). Specifically, in this study VIP+ cells were grouped into 21 different clusters. Notably, homologies in expression patterns were found in mouse visual cortex and lateral motor cortex, suggesting similar richness in VIP+ cell diversity across species and brain areas (Hodge et al., 2019). However, this may depend on the clustering method that one uses and the region being studied, since in a different RNA sequencing study conducted in the CA1 hippocampus, a smaller number of VIP+ cell clusters was found (Harris et al., 2018).

Importantly, some VIP+ cells, such as cholecystokinin co-expressing (CCK+) basket cells, primarily target excitatory cells, though they make up an apparently smaller fraction of VIP+ cells compared to I-S cells (Hájos et al., 1996; Kawaguchi and Kubota, 1996; Somogyi et al., 2004; Wang et al., 2004; Bezaire and Soltesz, 2013; Harris et al., 2018). Therefore, here we describe I-S cells generally as cells that preferentially inhibit other GABAergic interneurons. I-S cells, by this definition, have been described using imaging and electrophysiology techniques in several areas of the brain, including hippocampus (Gulyás et al., 1992, 1996; Acsády et al., 1996a,b; Tyan et al., 2014), frontal areas (Kawaguchi and Kubota, 1996, 1997; Pi et al., 2013), somatosensory cortex (Caputi et al., 2009; Lee et al., 2013; Prönneke et al., 2015), and visual cortex (Hajós et al., 1988; Zilles et al., 1991; Pfeffer et al., 2013). In this review we will highlight similarities in I-S cells that exist across these cortical areas.

In recent years, I-S cells with similar properties across several cortical areas have been characterized at multiple levels experimentally and inspired computational modeling work. Most commonly, I-S cells in various cortical areas have been studied *in vivo* through calcium imaging of VIP+ cells. This method of study alone however does not sufficiently distinguish VIP+ I-S cells from VIP+ non-I-S cells (i.e., such as VIP+ basket cells). As such, these studies can often lump together cells that inhibit pyramidal cells with those that disinhibit pyramidal cells. On the other hand, there has been much *in vitro* characterization work to complement the data obtained *in vivo*. In this review, we summarize previous work that has investigated and characterized the recruitment and impact of I-S cells found in various cortical areas of the brain. Ultimately, obtaining common principles in how disinhibitory I-S cells across cortical regions are involved in network computations will help inform disinhibitory strategies through which information is processed across the cortex.

## Hippocampal Formation

Characterization of interneurons in the hippocampal CA1 area (Freund and Buzsáki, 1996; Klausberger and Somogyi, 2008; Bezaire and Soltesz, 2013; Pelkey et al., 2017) has highlighted nuances in different forms of inhibitory control over inhibitory neurons beyond I-S cells (Chamberland and Topolnik, 2012). Based on synaptic density analysis, interneurons receive a substantial amount of inhibition relative to excitation (Gulyás et al., 1999), similar to pyramidal cells (Megias et al., 2001). As such, inhibition of inhibition can arise through several types of circuits, one of which is simply from the inhibitory neurons that target other inhibitory interneurons in addition to targeting pyramidal cells (Chamberland and Topolnik, 2012). Some examples include connections of neurogliaform cells to other inhibitory interneurons (Armstrong et al., 2012), basket cell connections to other basket cells (Cobb et al., 1997; Karson et al., 2009), recurrent connections between oriens lacunosum moleculare (OLM) and bistratified cells (Leão et al., 2012), and various OLM cell connections to interneuron dendrites in stratum lacunosum moleculare (SLM) (Katona et al., 1999). Inhibitory neurons in CA1 also receive long-range projecting GABAergic inputs (Chamberland and Topolnik, 2012). These include GABAergic projections from medial septum onto inhibitory interneurons in stratum oriens/alveus (SO/A) (Freund and Antal, 1988) and GABAergic projections from entorhinal cortex onto SLM interneurons (Melzer et al., 2012). In particular, axonal boutons from GABAergic medial septal projections form close appositions to PV+, CR+, calbindin- expressing (CB+), and CCK+ somata and dendrites, among others (Unal et al., 2015). Together with excitatory projections from entorhinal cortex and medial septum, this unveils a feedforward disinhibitory circuitry of specific CA1 pyramidal cell morphological compartments.

In the hippocampus, most I-S cells (19.4% of the interneuron population) express VIP, although some CCK+ basket cells (9.4% of the interneuron population) also express VIP—calculated in Bezaire and Soltesz (2013) according to Fuentealba et al. (2010) [though see Lorén et al. (1979), Köhler (1982), Léránth et al. (1984), Miettinen et al. (1992), Hájos et al. (1996), Tricoire et al. (2011)] for additional immunohistochemistry work investigating distribution densities of VIP+ cell types in hippocampus]. In the hippocampal CA1 area, I-S cells are divided into three types [I-S1, I-S2, and I-S3; Acsády et al. (1996a,b), Gulyás et al. (1996), Chamberland and Topolnik (2012), Tyan et al. (2014), Francavilla et al. (2015); Table 1], as well as a long-range projecting cell type that innervates the subiculum in addition to the hippocampal area CA1 (Francavilla et al., 2018). Because CCK+ basket cells also express VIP, isolation and manipulation of I-S cells is not straightforward, since results can possibly be contaminated with cells that input directly to pyramidal cells [e.g., see Pi et al. (2013), Fu et al. (2014), Pfeiffer and Foster (2015), Karnani et al. (2016a), Garcia-Junco-Clemente et al. (2017), Turi et al. (2019)]. Along these lines, combinatorial genetic-viral targeting methods could help further disambiguate I-S from non-I-S cell types (He et al., 2016).

**Table 1:** Hippocampal CA1 I-S cell types.

**I-S cell type**	**Somatic location**	**Dendritic profile**	**Axonal profile**	**Expression profile**	**Synaptic targets**
I-S1 cell	SO/A	SO/A	SO/A	VIP-/CR+	I-S1 cells
	SP	SP	SP		CB+ cells
	SR	SR	SR		VIP+ basket cells
		SLM	SLM		CR+ cells
I-S2 cell	SR	SR	SR	VIP+/CR-	CB+ cells in SR
	SLM	SLM	SLM		VIP+ cells in SR
					VIP+/CCK+ basket cells
I-S3 cell	SR	SR	SP	VIP+/CR+	OLM cells
	SP	SLM	SO/A	Penk+	Bistratified cells
	SO/A	SP		substance P receptor +	Basket cells
		SO/A		mGluR1α+	Axo-axonic cells
				COUP-TFII+	SO/A cells
				NOS+	
VIP-LRP cell	SO/A	SO/A	Subiculum	VIP+/CR+-	CA1:OLM cells
	SP	SP	SO/A	M2R+	Bistratified cells
	SR	SR	SP	CB+	CCK+ basket cells
	SLM	SLM	SR	CCK-	SC-associated cells
				NOS-	Subiculum:
				SOM-	Pyramidal cells
					Interneurons

*SO/A, Stratum Oriens/Alveus; SP, Stratum Pyramidale; SR, Stratum Radiatum; SLM, Stratum Lacunosum Moleculare; SC, Schaffer-Collateral; (+), expressing; (−), non-expressing; (+−), conditionally-expressing*.

The first type of I-S cells in the CA1, interneuron-specific 1 (I-S1) cells, have somata located in SO/A, pyramidale (SP), and radiatum (SR), express CR, and do not commonly express VIP (Klausberger and Somogyi, 2008; Chamberland and Topolnik, 2012; Harris et al., 2018) (Table 1). These primarily target CB+ cells, VIP+ basket cells, and CR+ cells, while at the same time avoiding PV+ basket and axo-axonic cells (Gulyás et al., 1996). Additionally, I-S1 cells are found to form dendro-dendritic and axo-dendritic connections with themselves with some indication of gap junction connections (Gulyás et al., 1996). The second type, interneuron-specific 2 (I-S2) cells, have somata present near the SR and SLM border, and express VIP, but not CR (Klausberger and Somogyi, 2008; Chamberland and Topolnik, 2012; Harris et al., 2018) (Table 1). These cells have different morphologies with axons that target CB+ and VIP+ cells in SR, including VIP+/CCK+ basket cells (Acsády et al., 1996a,b; Chamberland and Topolnik, 2012). Though I-S1 and I-S2 cells are thought to be VIP–/CR+ and VIP+/CR–, respectively, it is worth noting that more recently obtained transcriptomics data suggests a more nuanced expression profile (Harris et al., 2018). When clustering cell types according to transcriptomic expression profiles, it is observed that I-S1 cells (i.e., traditionally VIP-/CR+) show some expression of *VIP* and I-S2 cells (i.e., traditionally VIP+/CR-) show some expression of *Calb2* (i.e., the gene that codes for CR) genes relative to non-I-S cells (Harris et al., 2018). It is only when compared to each other, that these expression levels appear relatively low.

The third type (Table 1), are the interneuron-specific 3 (I-S3) cells, which co-express VIP and CR. These interneurons have cell bodies mostly within the SP and SR, with dendrites extending to SLM, and axons arborizing in the SO/A (Acsády et al., 1996a,b; Chamberland et al., 2010) (Table 1). Together with CR, I-S3 cells may co-express other neurochemical markers such as proenkephalin (Penk), substance P receptor, metabotropic glutamate receptor 1a (mGluR1α), COUP transcription factor 2 (COUP-TFII), and nitric oxide synthase (NOS) (Freund and Buzsáki, 1996; Blasco-Ibáñez et al., 1998; Ferraguti et al., 2004; Fuentealba et al., 2010; Tricoire et al., 2010). Electrophysiological characterization shows that I-S3 cells exhibit a high input resistance with irregular or regular spiking firing pattern (Chamberland et al., 2010; Tyan et al., 2014; Guet-McCreight et al., 2016). Also, it is known from dendritic calcium imaging experiments in combination with computational modeling that voltage-gated channels can be present in proximal dendrites of I-S3 cells (Guet-McCreight et al., 2016). In particular, there are proximal dendritic distributions of kinetically fast Kv3.1 channel subunits, which was confirmed using immunohistochemical analysis (Guet-McCreight et al., 2016). Furthermore, I-S3 cell distal dendrites receive excitatory input from entorhinal cortex via the temporoammonic pathway, while the proximal dendrites receive excitatory input from CA3 via the Schaffer collateral pathway (Luo et al., 2020). As well, a proportion of inhibitory inputs onto I-S3 cells are from I-S1, I-S2, and other I-S3 cells (Luo et al., 2020). I-S3 cells primarily form synapses onto SOM+ and mGluR1α+ OLM cells in SO/A (Chamberland et al., 2010; Tyan et al., 2014; Francavilla et al., 2015), but also contact bistratified cells, basket cells, putative axo-axonic cells, and various other SO/A interneuron types (Tyan et al., 2014). Compared to medial septal input to OLM cells, inhibitory currents generated by I-S3 cell input are smaller amplitude and have a slower time course (Chamberland et al., 2010). Despite this, optogenetic activation of CR+ cells, which includes the I-S1 and I-S3 cell types, at 5 and 10 Hz frequencies is sufficient to control the spike timing of OLM cells and to pace their activity at theta frequency (Tyan et al., 2014). Calcium imaging of activity of putative I-S3 cells *in vivo* showed that these cells tend to spike toward the end of theta-run epochs (Luo et al., 2020). Putative I-S3 cells in this study were identified through expression of VIP, somata located near the SP and SR border, and small somatic diameters (i.e., to distinguish them from VIP+/CCK+ basket cells, which have larger somatic diameters). Together with computational modeling and spike extraction analysis, it was found that I-S3 cells spike toward the rising to peak phases of theta waves, depending on the strengths of inputs from CA3 and entorhinal cortex (Luo et al., 2020). This capacity for phasic modulation suggests the involvement of I-S3 cells in the encoding and retrieval of information that occurs at distinct phases of theta waves (Siegle and Wilson, 2014). The VIP+/CR+ cells (i.e., putative I-S3 cells) that synapse onto SOM+ cells, including OLM cells, contain the α5 GABA_*A*_ receptor (α5-GABA_*A*_R). Interestingly, inhibiting α5-GABA_*A*_R at these synapses can modulate anxiety-like behaviors, with a possible impact on memory representations in the ventral hippocampus (Magnin et al., 2019).

In addition to the three I-S cell types described above, it is also worth mentioning a recently characterized long-range projecting (LRP) VIP cell type found in CA1 SO/A, with axons projecting to subiculum as well as to local interneurons in CA1 (Francavilla et al., 2018; Luo et al., 2019). VIP-LRP cells can have soma located in different CA1 layers, express muscarinic receptor 2 (M2R), and CB, and test negative for CCK and SOM (Table 1). In some cases, VIP-LRP cells with somata located in SP, SR, or SLM tested positive for Penk or CR suggesting further diversity, though the co-expression profiles of these markers in this cell type remain unknown. In the subiculum, VIP-LRP cells target both pyramidal cells and interneurons indiscriminately, while in the CA1, they target OLM, bistratified, and CCK+ basket cells and Schaffer-Collateral-associated cells. They are also electrically coupled with each other, but this mostly generates asynchronous spiking between pairs when depolarized past threshold using sinusoidal current injections. Further analysis revealed that this was because of low pass filtering properties which favored the conduction of slow after-spike hyperpolarizations, while attenuating the transfer of fast action potentials between electrically coupled cells. *In vivo*, these cells have elevated activity outside of theta-run periods, which could correspond with theta-off cells (Buzsáki et al., 1983; Colom and Bland, 1987). As well, they do not show activation during sharp wave-associated ripples. Although the inputs to these cells have not yet been characterized electrophysiologically, it is possible that they receive inhibitory input from I-S3 cells, since the somata of VIP-LRP cells were often decorated by VIP+ and CR+ axonal boutons (Francavilla et al., 2018). Though previous studies of I-S cells in hippocampus have generally classified I-S cells into these four types, a more recent transcriptomics study has also revealed a larger diversity of I-S cells in the CA1 area, with at least 8 transcriptomically-defined clusters [Harris et al. (2018); I-S1: 3 clusters; I-S2: 2 clusters; I-S3: 3 clusters]. Altogether, the characterization of I-S cell types in hippocampal CA1 area has highlighted a vast array of different cell types that are likely to enrich the mechanisms dedicated to information processing in this cortical area.

**Figure 1:**
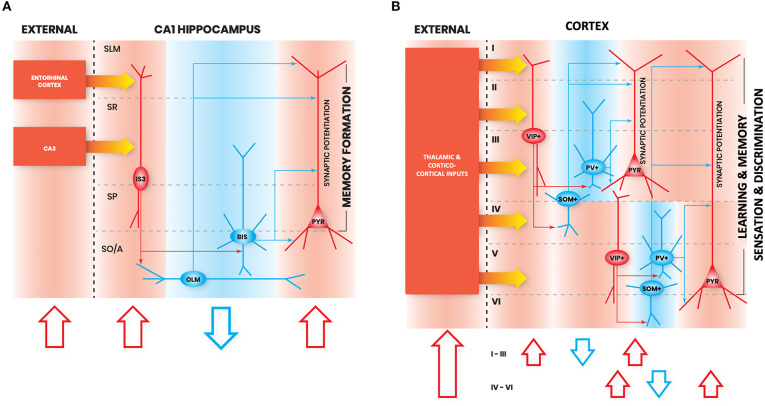
Interneuron-specific cell circuitry across cortical areas allows disinhibition and synaptic potentiation of pyramidal cells via external inputs. In these schematic figures, we highlight the commonalities in I-S circuitry across **(A)** CA1 hippocampus, and **(B)** neocortex. We chose to provide illustrations for these brain regions because enough details on I-S cell circuitry in these areas have been reported in the literature. Red shapes denote increased neural activation, and blue shapes denote decreased neural activation. The flow of each schematic is the following: increased activation of excitation from external inputs (1st red arrow) increases activation of I-S cells (2nd red arrow), which decreases activity of other local SOM+ and PV+ cell types (blue arrow), and allows an increase in gain in pyramidal cells (3rd red arrow). In principle, this can be generalized as the basic mechanism through which activation of I-S cells in different brain areas can lead to enhancements in learning, memory, and sensory discrimination. SO/A, Stratum Oriens/Alveus; SP, Stratum Pyramidale; SR, Stratum Radiatum; SLM, Stratum Lacunosum Moleculare; PYR, Pyramidal neuron.

Notably, there are not many studies to date that have examined different types of hippocampal CA1 I-S cells *in vivo* during active learning. However, there is a recent calcium imaging study looking at the activity of VIP+ cells in CA1 during head-restricted behavior (Turi et al., 2019), though this does not disentangle I-S cell types (Acsády et al., 1996a) from VIP+/CCK+ basket cells (Klausberger and Somogyi, 2008; Tyan et al., 2014; Francavilla et al., 2018; Luo et al., 2020). In this study, CA1 VIP+ cells exhibit a state-dependent disinhibition of pyramidal cells, which supports learning of reward site locations (Turi et al., 2019). During head-fixed locomotion, VIP+ cells were divided based on their activity pattern into those that are positively modulated by velocity, and those that are negatively modulated by velocity. Negatively velocity-modulated VIP+ cells exhibited activity either before or after running epochs, or both. Introduction of a spatially-guided reward learning task modulated the numbers of cells which falls into these functionally-defined VIP+ cell types. Interestingly, optogenetically suppressing VIP+ cell activity during this task led to an impairment in learning of the reward location and a decrease in the localization of pyramidal cell place fields near to the reward site, suggesting a role for VIP+ cells in learning the reward location and sharpening the memory representation. A network model further predicted that this sharpening of memory representation was due to the contribution of VIP+/CR+ cells, and not of VIP+/CCK+ basket cells (Turi et al., 2019). In line with this finding, another study found that chemogenetic silencing of CA1 VIP+ cells impairs spatial learning (Magnin et al., 2019). Together, these studies suggest an important role of I-S cells in learning and memory (Figure 1A).

## Frontal Areas

The spatial localization of I-S cells in frontal cortical areas can be extrapolated from the immunohistochemical studies that focused on the location of VIP+ cells. In rats, VIP+/CR+ cells show spatial distributions in layers II/III (i.e., 71% of CR+ cells are also VIP+) and layers V/VI (i.e., 94% of CR+ cells are also VIP+) (Kawaguchi and Kubota, 1997; Kawaguchi and Kondo, 2002). Smaller CCK+ cells tend to also be VIP+ and appear to be distributed similarly to VIP+/CR+ cells (Kawaguchi and Kubota, 1997). As well, some cells in layers II/III are immunoreactive for either VIP (i.e., 43% of VIP+ cells) or CR (i.e., 29% of CR+ cells), but not both (Kawaguchi and Kubota, 1997), similar to what is seen for hippocampal I-S1 and I-S2 cells. Further, VIP+ cells in frontal cortex, as classified based on their morphology, include bipolar cells, double bouquet cells, small basket cells, and arcade cells, of which small basket cells have rounder somata and smaller somatic diameters (Kawaguchi and Kubota, 1996; Kawaguchi and Kondo, 2002; Wang et al., 2002). In human frontal and temporal cortices, VIP+ cells can be found across all layers (Ong and Garey, 1991). In particular, VIP+ cells with small somatic diameters are located mostly in layers I and IV, whereas VIP+ cells with moderate somatic diameters are more numerous and more densely packed in layers II/III (Ong and Garey, 1991).

Notably, as far back as 2004, I-S cells were included in a network model of prefrontal cortex in order to make predictions on the neuronal operations that support working memory (Wang et al., 2004). Here, I-S cells were proposed to contribute to working memory by suppressing the dendrite-targeting interneuron activity during sensory inputs, thus creating inverted sensory tuning curves in dendrite-targeting interneurons. Otherwise, dendrite-targeting interneurons provided resistance against distracting stimuli through elevated spontaneous activity. This pathway gating/ungating roles of I-S cells is further reviewed in Wang and Yang (2018).

Similar to I-S3 cells in CA1, VIP+ cells in auditory and medial prefrontal cortices primarily inhibit SOM+ cells, as well as a fraction of PV+ cells (Pi et al., 2013). A small percentage also contact pyramidal cells, which could represent an overlap with non-I-S VIP+ cells. *In vivo*, VIP+ cells in these areas are strongly but transiently activated following punishments in a go/no-go auditory discrimination task, and show a weaker but sustained response during rewards (Pi et al., 2013). Also, VIP+ cells are similar to pyramidal cells in that they exhibit responses during both go and no-go trials, while SOM+ and PV+ cells exhibit a response bias toward go trials (Kamigaki, 2019a).

In dorsomedial prefrontal cortex (also reviewed in Kamigaki, 2019a,b), activation of PV+ or SOM+ cells during this type of go/no-go task impairs performance, while activation of VIP+ cells enhances performance (Kamigaki and Dan, 2017). Similar findings were seen in a delayed two-alternative forced-choice task, which suggests an importance for VIP+ cell activation in enhancing short-term memory retention (Kamigaki and Dan, 2017). In addition, disinhibition through activation of VIP+ cells in prefrontal cortex enhances theta rhythm synchrony and communication from hippocampus to prefrontal cortex (Lagler et al., 2016; Lee et al., 2019). For example, in an elevated plus maze task, animals display avoidance of the open arm component of the maze, and this is accompanied with VIP+ cell activation and disinhibition of responses to hippocampal inputs, which can carry anxiety related-information (Lee et al., 2019). Correspondingly, inhibition of VIP+ cells leads to reduced open arm avoidance when there is theta synchrony between the two regions. During a different and more complex cue-matching-to-place task, the activity of PV+ basket cells in the prelimbic area of the prefrontal cortex forms neural ensembles that are either activated or suppressed at different stages of the task, thus allowing “multi-layered cognitive computations” (Lagler et al., 2016). More specifically, the activation or suppression of individual basket cells is correlated to the amount of VIP+ cell inputs that they receive.

In frontal association cortex *in vivo*, feedforward inhibition from VIP+ cells creates a pull-push circuitry, alternatively to the classical push-pull circuitry that can be generated through feedforward excitation (Garcia-Junco-Clemente et al., 2017). This is observed since VIP+ cells in this brain area make both direct connections to pyramidal cells in addition to connections to SOM+ cells. Without further characterization of VIP+ cell types in this brain area however, it is difficult to say whether this arises from VIP+ cell uniqueness in frontal association cortex, or different I-S and non-I-S VIP+ cell types.

In summary, from work done in frontal areas, it is clear that I-S cells are involved in enhancing learning and memory discrimination, are recruited during theta rhythms, and primarily target SOM+ cells, but also PV+ cells (i.e., similar to hippocampal CA1 I-S3 cells; Figures 1A,B). Further characterization of VIP+ cell types in frontal areas is needed to distinguish how much I-S cell types in these areas contact pyramidal cells, compared to non-I-S VIP+ cells.

## Somatosensory Cortex

In the somatosensory barrel cortex, VIP+ cell dendrites often exhibit bipolar or tufted morphologies and span across cortical layers and columns (Bayraktar et al., 2000; Prönneke et al., 2015). Particularly, in layer IV, VIP+ and SOM+ cells, but not PV+ cells, show a higher density within intercolumnar septal areas (Bayraktar et al., 2000; Almási et al., 2019). Moreover, different VIP+ cell morphologies can be found across all layers in barrel cortex, though they exhibit a higher density in layers II/III (Prönneke et al., 2015; Almási et al., 2019). In a study of CR+ cells spanning across cortex, it was also found that CR is generally expressed in bipolar and multipolar cells (Caputi et al., 2009). While both preferentially target other interneurons, electrophysiological characterization of these cells in barrel cortex demonstrated key excitability and synaptic differences—most notably the existence of asymmetric electrical connections between multipolar CR+ cells and multipolar PV+ cells (Caputi et al., 2009).

Also in the somatosensory barrel cortex, VIP+ cells receive inputs from the primary vibrissal motor cortex pyramidal cells, and preferentially inhibit SOM+ cells (Lee et al., 2013). This highlights another cortical disinhibitory circuitry, where during whisking behavior *in vivo*, VIP+ cells show enhanced activation, while SOM+ cells reduce their activity (Gentet et al., 2012; Lee et al., 2013; Yu et al., 2019). This finding was both layer-specific (i.e., VIP+ cells in layers II/III) and behavior-dependent, where it was specifically observed during active wakefulness voluntary whisking (Yu et al., 2019). When tested in a goal-directed behavioral context where mice were actively attempting to localize an object, whisker touch events slightly suppressed VIP+ cells, with an immediate activation of fast spiking cells and a delayed activation of SOM+ and excitatory cells (Yu et al., 2019). VIP+ cells have also been shown to contact CB+ cells (Staiger et al., 2004) as well as PV+ cells (Dávid et al., 2007), where higher densities of VIP+ cell inputs to PV+ cells were associated with higher densities of excitatory inputs. Axon terminals from VIP+ cells in somatosensory cortex also highly express mGluR7a, a metabotropic glutamate receptor, which can be expressed in axon terminals that contact SOM+ and PV+ cells (Dalezios et al., 2002). As well, VIP+ cells can also target excitatory cells (Zhou et al., 2017), though it is unknown if this is due to different VIP+ cell types, or specialized layer-specific connections across the cortical layers of barrel cortex. In addition to inputs from the primary vibrissal motor cortex, VIP+ cells in somatosensory barrel cortex generally also receive inputs from thalamus, and other cortical areas [i.e., proportionally more inputs than other inhibitory cell types receive; Wall et al. (2016)], in a spatially-specific way (Sohn et al., 2016; Almási et al., 2019). For example, when preferentially stimulating either the layer IV-projecting first-order ventral posterior medial thalamic nucleus or the layer I and V-projecting higher-order posterior thalamic nucleus, VIP+ cells are more broadly activated across layers when compared to the excitatory cells, PV+ cells, and SOM+ cells (Sermet et al., 2019). This is consistent with their bipolar dendritic arbors, which span across layers and can allow VIP+ cells to receive inputs that arrive to different cortical layers. In line with this, cortico-cortical and thalamo-cortical inputs in barrel cortex preferentially target the distal dendritic compartments of VIP+ cells (Sohn et al., 2016). Likewise, SOM+ cell inhibitory inputs also preferentially target distal dendritic compartments of VIP+ cells, while PV+ cell inhibitory inputs target the perisomatic compartments of VIP+ cells (Sohn et al., 2016). Higher-order thalamic inputs to VIP+ cells in particular are critical to the induction of LTP in disinhibited layers II/III pyramidal cells during whisker stimulation (Williams and Holtmaat, 2019).

Furthermore, following the study of Lee et al. (2013), it was found that the activity of SOM+ cells in barrel cortex during whisking depends highly on the cortical layer and the SOM+ cell morphology (Muñoz et al., 2017). In particular, some whisking-suppressed SOM+ cells presumably receive stronger inhibition from VIP+ cells during whisking, which, when removed, causes a reversal in their responses to whisking behavior. Blocking cholinergic receptors with bath application of atropine further highlights a state-dependent response, since all SOM+ cells become suppressed during whisking following this manipulation (Muñoz et al., 2017). In barrel cortex, there are also some data on the synaptic inputs by VIP+ and PV+ cells onto Martinotti cells, a SOM+ cell type that is analogous to OLM cells in the hippocampus (Walker et al., 2016). Specifically, Martinotti cells receive a stronger inhibition from PV+ cells. As well, PV+ cell inputs show a frequency-invariant short-term depression, while VIP+ cell inputs exhibit short-term facilitation. This suggests two temporally-specific modes of control over Martinotti cell spiking that can coordinate the disinhibition of pyramidal cells.

In barrel cortex and entorhinal cortex *in vitro*, VIP+ cells also show differential activation according to brain states (Neske et al., 2015; Neske and Connors, 2016). While PV+ cell activity is dominant during up states, there is also considerable spiking from SOM+ and VIP+ cells in barrel cortex, though spiking from these cells during up states is considerably smaller in the entorhinal cortex (Neske et al., 2015). Surprisingly, while optogenetic silencing of SOM+ cells enhances pyramidal cell excitability during up states, optogenetic activation or silencing of VIP+ cells in barrel cortex does not have any effect on pyramidal cell spiking (Neske and Connors, 2016). This suggests the existence of dynamically modulated states where VIP+ cells will not significantly impact network activity, despite their inhibitory connections to SOM+ cells.

VIP+ cells in barrel cortex are also extensively characterized *in vitro* (Prönneke et al., 2015). This work demonstrates the existence of layer-specific distributions of VIP+ cell morphologically-defined types as well as a variety of different electrophysiologically-defined types, which do not necessarily map onto each other (Prönneke et al., 2015). In fact, a large diversity of different neuronal types could be present in the VIP+ cell population in barrel cortex. Furthermore, certain VIP+ cells in barrel cortex are sensitive to depolarization-inducing neuromodulation from acetylcholine or serotonin, which can effectively switch their firing patterns (Prönneke et al., 2019). Similarly, in auditory cortex, VIP+ cells are very sensitive to nicotinic neuromodulation, where bath application of nicotine can induce large depolarizations in VIP+ cells, with consequential weak depolarizations in pyramidal cells (Askew et al., 2019).

In summary, similar to I-S cells in other areas, I-S cells in somatosensory cortex appear to also primarily target SOM+ cells. Particularly, they target Martinotti cells, which exhibit properties similar to OLM cells, the primary synaptic target of I-S3 cells in the hippocampus. As well, I-S cells in somatosensory cortex receive spatially organized inputs, which appears to be the case for I-S cells in other areas as well. Altogether, given the similarities in activation and circuitry between I-S cells across different cortical areas, it appears that they may provide similar contributions to network function, such as induction of synaptic plasticity in pyramidal cells during behavior (Figure 1B).

## Visual Cortex

In visual cortex, VIP+ cells have somata located in layers II/III, with dendrites distributed in layers I and II, and axons innervating layers I/II, IV, and VI (Hajós et al., 1988; Zilles et al., 1991; Ji et al., 2016). Notably, in both visual and auditory cortices, despite a higher somatic density in layers II/III, only VIP+ cells with somata in layer IV are likely innervated by thalamocortical inputs (Ji et al., 2016), which may be due to differences in laminar dendritic branching patterns between layers II/III and layer IV VIP+ cells or electrotonic distances from soma.

Furthermore, in visual cortex, the activity patterns of VIP+ cells *in vivo* are similar to PV+ cells in that they exhibit strong intra-population coupling (Knoblich et al., 2019). Similar to other areas, visual cortical VIP+ and SOM+ cells inhibit each other bidirectionally, and both SOM+ and VIP+ cells receive non-overlapping inputs from layers II/III pyramidal cells (Pfeffer et al., 2013; Karnani et al., 2016a,b). Moreover, ErbB4 (i.e., a tyrosine kinase receptor associated with signaling factor Neuregulin-1) in VIP+ cells regulates these connections throughout development (Batista-Brito et al., 2017). Specifically, ErbB4 regulates both VIP+ cell targeting of SOM+ cells, as well as excitatory synaptic targeting of VIP+ cells (Batista-Brito et al., 2017). In fact, abolishing ErbB4 in VIP+ cells alters network dynamics and leads to a loss in visual response selectivity and impaired sensory learning (Batista-Brito et al., 2017). *In vivo*, locomotion enhances VIP+ cell activation, which causes an increase in gain in visual responses (Fu et al., 2014), and modulation of cortical plasticity (Fu et al., 2015). This occurs through inputs from the basal forebrain to VIP+ cells, and is independent of visual stimulation (Fu et al., 2014). This disinhibitory circuitry is context-dependent where during visual stimulation, all of VIP+, PV+, and SOM+ cells in layers II/III and IV show locomotion-associated activation. This indicates that SOM+ cells are activated by visual stimulation during movement, despite inhibition from VIP+ cells (Pakan et al., 2016). In darkness, VIP+ and PV+ cells remain locomotion-associated, while SOM+ cells become silent.

VIP+ cells in visual cortex *in vivo* are also active during non-locomotion, visual stimulation, and under anesthesia (Jackson et al., 2016). Specifically, two-photon calcium imaging has shown that VIP+ cell activity correlates most with the activity of pyramidal cells, and plays a causal role in generating states of high excitatory activity (Jackson et al., 2016). More specifically, suppression of VIP+ cells leads to a reduction of spontaneous excitatory network activity across different behavioral states (Jackson et al., 2016). This directly plays a role in visual spatial frequency tuning of pyramidal cells, where activation of VIP+ cells generates responses to higher visual spatial frequencies and inactivation of VIP+ cells generates responses to lower visual spatial frequencies (Ayzenshtat et al., 2016). During visual stimulation, VIP+ cells also respond differently to novel vs. familiar images (Garrett et al., 2020). While they become activated during novel images, they are suppressed during familiar images. As well, VIP+ cell activation ramps up when expected visual stimuli are omitted during sequences of visual stimuli (Garrett et al., 2020). Similarly, activation of VIP+ cells during visual stimulation (i.e., using a contrast drifting Gabor stimulus) enhances contrast detection, whereas activation of PV+ or SOM+ cells during the visual stimulation reduces contrast detection (Cone et al., 2019).

Interestingly, VIP+ cells in visual cortex following animal exposure to light exhibit a high expression of insulin like growth factor 1 (IGF1), an experience-regulated gene (Mardinly et al., 2016), which is in contrast to PV+ and SOM+ cells. Over-expression of IGF1 also promotes inhibitory inputs to VIP+ cells, thus reducing their activity, and, accordingly, the disinhibition of pyramidal cells. Additionally, when testing ocular dominance plasticity, knocking down IGF1 increases disinhibition and enhances visual acuity in an experience-dependent manner (Mardinly et al., 2016).

Visual acuity is also potentially modulated by inputs from the cingulate area in frontal cortex, which improves visual discrimination via strong connections to VIP+ cells in visual cortex, in addition to contacting PV+ cells, SOM+ cells, and pyramidal cells (Zhang et al., 2014). Particularly, focal layer-specific activation of cingulate area axons in visual cortex leads to SOM+ cell-mediated surround suppression and VIP+ cell-mediated center facilitation (i.e., disinhibition) (Zhang et al., 2014). Based on computational modeling work, these types of connectivity mechanisms can sharpen and enhance visual responses for optimal encoding of visual stimuli (Lee and Mihalas, 2017; Lee et al., 2017). Moreover, VIP+ cells *in vivo* possess vertically elongated axons with narrow spatial layouts that allow lateral disinihibition of pyramidal cells, which can generate local transient holes in the blanket of inhibition in visual cortex (Karnani et al., 2014, 2016a).

In visual cortex, there is also some indication that VIP+ cells may be involved in associative learning through long-range top-down projections from the retrosplenial cortex (Makino and Komiyama, 2015). Here it was shown that retrosplenial inputs to layers II/III pyramidal cells become enhanced during a visually-guided active avoidance task, where mice are trained to run on a treadmill when they see a drifting grating stimulus with particular orientation. Comparatively, bottom-up pyramidal cell projections from layer IV and SOM+ cell projections from layers II/III (i.e., which gate retrosplenial inputs) become weaker over the course of this task. Both retrosplenial inactivation and SOM+ cell activation were sufficient to reverse the learning-related changes in layers II/III. Although no changes in VIP+ cell activation were observed throughout this task, it is nonetheless possible that VIP+ cells are entrained by retrosplenial inputs to suppress SOM+ cells, which would ungate retrosplenial inputs to layers II/III pyramidal cells. This, however, remains to be tested directly.

Once again, like I-S cells in the hippocampus, frontal areas, and somatosensory cortex, I-S cells in visual cortex exhibit similar morphological properties and primarily target SOM+ cells. Similar to I-S cells in other areas, visual cortical I-S cell activation contributes through enhancement of sensory discrimination and by promoting plasticity mechanisms, which, again, suggests similar roles for I-S cells across cortical areas (Figure 1B).

## Motor Cortex

VIP+ cell morphological and electrophysiological characterization has also been performed in the motor cortex. Here I-S cells also tend to have bipolar morphologies with irregular spiking activity and local intracortical glutamatergic inputs from pyramidal cells (Cauli et al., 1997; Porter et al., 1998). They are further characterized as two distinct populations, based on differences in burst duration, and expression of CR and choline acetyltransferase.

In motor cortex, VIP+ cells are also involved in motor skill learning (Adler et al., 2019). In this study, mice were trained to run forwards or backwards at fixed speeds on a treadmill, effectively changing their gait patterns such that they run with a structured pattern. During this motor learning, layers II/III pyramidal cells exhibited sequential activation patterns across the population that are shifted compared to normal treadmill running—a finding that was dependent on the presence of CaMKII-mediated synaptic plasticity (Adler et al., 2019). SOM+ cells, on the other hand, exhibited diverse responses where they would become enhanced, suppressed, or unchanged at the onset of forward and backward running. As well, their responses to either forward or backward running was not indicative of what their response would be in the opposite case. Interestingly, activation of SOM+ cells suppressed both the temporal shift in the sequential activation of pyramidal cells as well as motor learning. Conversely, suppressing SOM+ cells during new learning de-stabilized previously learned motor skills (i.e., when switching from a forward running to backward running learning paradigm). Further tests revealed that VIP+ cells, which become activated during both forward and backward running, are necessary for the shifted sequential pyramidal cell activation patterns and motor learning to occur, but not necessary for preserving previously learned motor skills. Altogether, Adler et al. (2019) provides a clear demonstration of how VIP+ cells can inhibit SOM+ cells, causing disinhibition in pyramidal cells and allowing synaptic plasticity and learning to occur.

## Basolateral Amygdala

Although it is not a part of cerebral cortex, certain commonalities also exist with VIP+ cells located in the basolateral amygdala. In this area, VIP+ cells have been classified as either I-S or cannabinoid-expressing basket cells (Rhomberg et al., 2018). Additionally, three types of activity patterns are observed when recording from I-S cells, based on the number of spikes observed upon depolarization. Here, I-S cells primarily target other I-S cells, CCK+ basket cells, and neurogliaform cells. I-S cells themselves receive a dense inhibition, of which only a small proportion is from other I-S cells (Rhomberg et al., 2018). Though connections to SOM+ or PV+ cells were not tested in this study, other studies showed that VIP+ cells target CB+ cells, which include SOM+, PV+, and CCK+ cells (Muller et al., 2003; Krabbe et al., 2018). More recently, deep-brain calcium imaging and optogenetic experiments have demonstrated the involvement of the basolateral amygdala VIP+ cells in associative learning (Krabbe et al., 2019). More specifically, these cells were activated by aversive stimuli, were modulated by expectations, and were necessary for the induction of synaptic plasticity and learning to occur. Moreover, VIP+ cell contribution to circuit function is achieved through innervation of SOM+ and PV+ cells, which allows disinhibition of projecting neurons in basolateral amygdala (Krabbe et al., 2019). Overall, this circuitry motif and functional role are similar to the VIP+ cell circuitry and function seen in other brain areas.

## I-S Cells as a Clinical Target?

Given their specialized connectivity, disinhibitory control, and potentially beneficial effects on learning and memory, it is worth considering I-S cells as potential targets in clinical studies. As such in this section we will give an overview of some studies that have already investigated I-S cells in various neuropathologies and clinical contexts.

In the CA1 hippocampus, I-S3 cells have been studied in the context of status epilepticus using a mouse model of temporal lobe epilepsy, where these cells showed altered dendritic morphologies and passive membrane properties, and provided a lower inhibition to their postsynaptic targets (David and Topolnik, 2017). Although I-S3 cell densities were preserved in this study, it has been shown in human dentate gyrus that densities of CR+ cells are reduced in epilepsy (Maglóczky et al., 2000). Furthermore, hippocampal CR+ cells have altered morphologies and connectivity to a degree which depends on the severity of the temporal lobe epilepsy (Tóth et al., 2010; Thom et al., 2012). In another mouse model of epilepsy desynchronization of interneuron firing between regions CA1 and dentate gyrus lead to destabilization of CA1 place cell Shuman et al. (2020). As well, in an optogenetically-induced epilepsy model, it is observed that different motor cortex neuron types follow different trajectories throughout a seizure (Khoshkhoo et al., 2017). While PV+, SOM+, and VIP+ cells all become activated at the onset of a seizure, pyramidal cells exhibit a delay in activation. As well, PV+ and SOM+ cells exhibit a gradual increase in activation throughout the seizure, while VIP+ cell activation peaks near the mid-point of seizures. Interestingly, optogenetic inhibition of VIP+ cells consistently disrupts seizure onset, and reduces the duration of seizures, which highlights VIP+ cells as a potential neuromodulatory target for seizure control (Khoshkhoo et al., 2017). Along these lines, VIP+ cells, alongside PV+ and SOM+ cells in motor cortex, are known to be manipulable through brain machine interfacing (Mitani et al., 2018).

Somatosensory cortex VIP+ cells have also been studied in a mouse model of neuropathic pain. Specifically, VIP+ cells are over-active during neuropathic pain, which parallels the activity of pyramidal cells, but is in contrast to a reduced activity of PV+ and SOM+ cells (Cichon et al., 2017). This combination of effects therefore leans in favor of pyramidal cell hyper-excitability through disinhibition. Moreover, activation of SOM+ cells was sufficient for suppressing pyramidal cell hyperactivity and reversing symptoms of neuropathic pain.

VIP+ cells can also contribute to the pathogenesis of Dravet syndrome, a neurodevelopmental disorder associated with a loss of functional variants of the gene SCN1A that encodes for the Nav1.1 channel subunits (Goff and Goldberg, 2019). More specifically, a mouse model of Dravet syndrome (*Scn*1*a*^+/−^) had previously been associated with a loss of excitability in PV+ and SOM+ cells, which both express Nav1.1 (Yu et al., 2006; Ogiwara et al., 2007; Tai et al., 2014; De Stasi et al., 2016; Favero et al., 2018). In addition, in *Scn*1^+/−^ mouse somatosensory and visual cortices, VIP+ cells that displayed irregular spiking, but not those that displayed continuous adapting spiking, were found to exhibit reductions in gain (Goff and Goldberg, 2019). Notably, the irregular spiking firing pattern did not coincide with whether VIP+ cells co-expressed CR or CCK, nor with whether the morphology was bipolar vs. multipolar. These irregular spiking VIP+ cells were also found to express Nav1.1, which explains their involvement in the pathogenesis, and the irregular spiking pattern was dependent on the activation of the M-type potassium channels (Goff and Goldberg, 2019).

CR+ cells have also been studied in the context of an Alzheimer's disease mouse model where decreased numbers were reported and, in parallel, increased amyloid beta deposits were observed within the axonal fields of CR+ cells (Baglietto-Vargas et al., 2010). These were distinguished from other Cajal-Retzius cells, which also express CR but did not show decreased numbers. However, reduced densities of CR+ cells have not been observed in human dentate gyrus or entorhinal cortex, and these do not appear to co-localize with neurofibrillary tangles, nor their axonal fields with amyloid beta plaques (Brion and Résibois, 1994). On the other hand, CR+ cells in this study did exhibit reduced dendritic trees and dystrophic fibers in subjects with Alzheimer's disease (Brion and Résibois, 1994). During aging, there is also a down-regulation of genes associated with synaptic transmission in SOM+ and VIP+ cells in human frontal cortex, suggesting age-dependent changes in the functionality of this circuitry (French et al., 2017). As such, stimulating this circuitry (e.g., pharmacologically exciting VIP+ cells) could be a viable option for targeting age-related changes in cognition, such as learning and memory.

## Summary and Conclusions

Overall, it is clear from this body of literature that I-S cells across cortex share many common principles in their organization, and I-S cell types may be particularly diverse. We highlight this with the illustrations shown in Figure 1. Of course, much more information on the detailed I-S circuitry organization is currently available for CA1 hippocampus when compared to other cortical areas, which often characterize cell types through neurochemical marker expression alone. Amongst commonalities we note the tendency of I-S cells to have vertical bipolar-shaped morphologies with dendrites that project across layers. This allows them to receive inputs from multiple layer-specific external projections. We also indicate commonalities in their preferential targeting of SOM+ cells (and PV+ cells to a lesser extent), as well as their ability to disinhibit pyramidal cells through this circuitry, which may create a window for synaptic plasticity to occur. Altogether, these features allow I-S cells across cortex to perform a wide array of different cortical computations during behavior. Because of their common ability to effectively gate synaptic plasticity in pyramidal cells across cortical structures, I-S cells in cortex offer an interesting therapeutic target for neuromodulation by using drugs that specifically modulate the excitability of I-S cells. For example, we can study how global enhancement or suppression of I-S cell activity across the cortex might impact behavior. Along these lines, since similar analogous circuitries exist across the cortex, it seems that I-S cells in general play a role in learning and sensory tuning of pyramidal cell receptive fields. As such, activation of I-S cells across cortical structures *in vivo* offers a target for globally enhancing performance on memory-related tasks.

It is important to note that VIP+ cells exhibit a large diversity in transcriptomic, morphological and physiological properties (Prönneke et al., 2015; Harris et al., 2018; Gouwens et al., 2019; Hodge et al., 2019), which so far made the definition of cell types largely impossible. While I-S cells exhibit commonalities across different cortical structures, these cells might thus still exhibit local differences in functional roles and contributions to network activity. Moreover, different balances of I-S vs. non-I-S VIP+ cells across different brain areas could give the appearance of different region-specific functional contributions to circuit function *in vivo*, when in fact it only appears this way because I-S cells are not sufficiently isolated from non-I-S cells when VIP+ cells are targeted (e.g., CCK+/VIP+ basket cells). Further classification studies based on transcriptomic and morphological characteristics in relation to synaptic connectivity (i.e., inputs and outputs) across different cortical areas would help to elucidate these aspects of I-S cell diversity.

Another aspect to highlight from these studies is that I-S cells from any particular region appear to receive a mixture of inputs from different distant regions. It has already been postulated (Wang and Yang, 2018), and demonstrated in a model (Yang et al., 2016), that the reason for this is because I-S cells can gate particular external pathways and allow flexible switching between information arriving from different external structures. As such, without I-S cell contributions to circuit function throughout the brain, it is possible that information would not be properly routed during behavioral tasks where integration of different types of sensory stimuli are relevant. This is particularly relevant in Lagler et al. (2016) where basket cell activation during a complex behavioral task was dependent on the level of I-S cell activation, suggesting the existence of complex learned disinhibitory pathways.

To conclude, we present in this review compelling evidence indicating that I-S cells across different cortical areas share many morphological, physiological, and connectivity features, which allows them to contribute in similar ways to network function. As such, we highlight that I-S cells across cortex are a crucial network component that is necessary for supporting sensory discrimination, learning, and memory formation.

## Author Contributions

AG-M: conceptualization, visualization, writing—original draft preparation, and writing—review and editing. FS and LT: project administration, resources, supervision, and writing—review and editing.

## Conflict of Interest

The authors declare that the research was conducted in the absence of any commercial or financial relationships that could be construed as a potential conflict of interest.
